# Dynamic Moduli of Polybutylene Terephthalate Glass Fiber Reinforced in High-Temperature Environments

**DOI:** 10.3390/ma14030483

**Published:** 2021-01-20

**Authors:** Carmelo Gómez, Jorge Mira, F.J. Carrión-Vilches, Francisco Cavas

**Affiliations:** 1 Doctorate Program in Industrial Technologies, International School of Doctorate, Technical University of Cartagena, 30202 Cartagena, Spain; gomezgarciacarmelo@gmail.com (C.G.); miraperezjorge@gmail.com (J.M.); 2 Materials Science and Metallurgical Engineering Group, Materials Engineering and Manufacturing Department, Technical University of Cartagena, 30202 Cartagena, Spain; fjc.vilches@upct.es; 3 Department of Structures, Construction and Graphical Expression, Technical University of Cartagena, 30202 Cartagena, Spain

**Keywords:** polymer composites, storage modulus, glass fiber, high temperature

## Abstract

The aim of this work was to show the evolution over time of the dynamic moduli in components made of Polybutylene Terephthalate reinforced with glass fiber when they are held to temperatures close to the glass transition temperature over time. For this purpose, PBT samples reinforced with short, glass fibers of Ultradur^®^ material with 0%, 20%, and 50% in weight content were tested. Dynamic moduli showed an increment with glass fiber content showing a nonlinear behavior with the temperature. The evolution of storage modulus was depicted by means of a modified law of mixtures with an effectiveness factor depending on temperature and fiber content, whereas the evolution over time was obtained with a time–temperature transformation generated with the TTS Data Analysis software of TA-instruments for a given temperature. Storage modulus showed a linear relationship with glass fiber content when components were held to temperatures near to their respective glass transition temperature, obtained from the maximum of loss modulus curve with temperature. In summary, the value and evolution of dynamic moduli of PBT samples improved with glass fiber content, allowing us to increase the durability of components when they are submitted to high-temperature environments.

## 1. Introduction

Overcoming new technological challenges and greater social awareness of environmental protection are the ideal basis for investigating new materials that improve the properties of existing ones, but by putting natural resources to better use. In recent years, considerable efforts have been carried out to develop biodegradable materials [1,2,3,4,5,6,7,8] and composites [9,10], allowing the reduction of plastic contamination and optimizing composite components and structures. Thermoplastic materials have shown a strong growth due to their good mechanical properties and possibility to be recycled [11]. Furthermore, properties can be amended by using additives or blending different polymers.

Polybutylene terephthalate (PBT) is a thermoplastic polymer with excellent dimensional stability, is very rigid, and offers good thermal resistance, electric isolation, and few dielectric losses. The increasing use of this material is related mainly with the electronic sector, particularly with the incorporation of new functionalities into the automotive sector by the integration of connectors and sensors due to its good performance in micro-molding processes [12] and 3D printing technology with good thermal and mechanical properties [13,14].

PBT offers extremely good versatility when mixed with other materials, which has enabled additives to be incorporated, such as flame retardants, and particularly more enviro-friendly, nonhalogen additives to improve their properties against fire [15,16,17,18,19,20,21].

PBT components are mainly manufactured by injection methods in which short, glass fibers are incorporated as reinforcement. The reinforced material obtained can be shredded and injected in a further process maintaining good mechanical properties [22]. PBT components show good strength and stiffness properties but also poor performance as regards impact and fracture mechanisms. Material properties can be improved adding new reinforced materials [5,23] or blended with other polymer materials.

Incorporating elastomeric materials increases fracture tenacity, performance upon impacts, and components’ surface resistance by, in turn, increasing the retention and recovery capacities of the material’s properties, even after aging processes [24,25,26,27]. The improvement is attributed to local deformation zones being produced, which act as energy dissipaters and would, thus, increase the plasticity of the analyzed samples.

Improving the properties linked with impacts makes resistance to flexion and storage modulus worse. Zhang et al. [28] ran tests with thermoplastic polyurethane (TPU). Dynamic mechanical analysis (DMA) indicated that the addition of TPU increases tensile and notched Izod impact strength with a small reduction in flexural strength and modulus. Other authors have demonstrated that when other materials were also added, such as PTW poly(ethylene-butylacrylate-glycidyl methacrylate copolymer), they allowed further increments in the storage modulus and the loss factor tangent, although performance worsened after a certain percentage [29].

Strength and stiffness of polymers’ materials depend on the strain rate. Abdo et al. [30,31] and Isogai et al. [32] incorporated thermoplastic polyester elastomers (TTPE) into short, glass fiber-reinforced PBT samples in order to investigate the strain speed dependence. The results indicated that ultimate tensile strength is sensitive to strain rate. Both damage initiation and crack growth were affected by blending with TTPE.

Stiffness of polymer materials linearly increases with the strain rate logarithm [33]. This is because polymer chains are less prone to relaxation mechanisms. The material is less ductile and offers greater resistance and a higher modulus of elasticity, while properties worsen with temperature and presence of liquids [34].

Incorporating other polymer materials also allows dynamic performance to increase, and, hence, the fatigue properties of the obtained composite material also increase. Varga and Bartha [35] demonstrated improved dynamic properties for short, glass fiber-reinforced PBT after incorporating cyclic oligomers (CBT-100), and the fatigue performance analysis indicated differences with conventional metal-type materials. Eftekhari et al. [36] tested fatigue with variable amplitude, and a rigidity process in the first load cycle was observed. Schaaf et al. [37] developed a method to estimate the life cycle of short, glass fiber-reinforced polymers, whereas Mbyniec and Uhl [38] proposed a failure criterion that depended on both aging time and fibers’ alignment. The aim of these proposals was to analyze the durability and resistance of materials.

Durability of polymers is strongly dependent on thermal aging, with action of elevated temperatures being a key factor in electrical components submitted to high-temperature environments during long periods of time. Hashemi [39] investigated the behavior of PBT reinforced with short, glass fibers in samples between 23 °C and 100 °C. The results showed that tensile strength and elastic modulus decreased nonlinearly when temperature was increased. Mortazavian and Fatemi [33] analyzed the performance of glass fiber-reinforced PBT and PA6 samples. A Ramberg–Osgood-type relationship was used to relate stress and strain. Time–temperature superposition principle with a shift factor of Arrhenius types was applied to obtain tensile strength data at different temperatures.

PBT is an ideal candidate in the growing number of electronic and electrical components in the transport sector, such as batteries and components for chargers, due to its excellent properties as an electric insulator.

These components could be submitted to high-temperature environments during long periods of time, and stiffness and energy absorption are key factors due to dimensional stability and durability requirements.

This work aimed to analyze the influence of glass fiber content on the evolution with temperature and frequency of the dynamic moduli. By means of a time–temperature transformation, the evolution with time of dynamic moduli was obtained. For this purpose, temperatures that came close to the material’s glass transition temperature were considered.

## 2. Experimental

### 2.1. Materials and Sample Preparation

PBT samples were manufactured with three variants of Ultradur^®^ material obtained from the company BASF (Ludwigshafen, Germany). B 4520 variant was unreinforced, whereas the variants B 4300 G4 and B4300 G10 contained 20% and 50% of glass fiber reinforcement in weight equivalent to 11.7% and 34.7% percentage in volume, respectively. To manufacture samples, a DEU 250H55 mini-VP injection machine (Barcelona, Spain) was employed by applying 12 MPa injection pressure and 9 MPa maintenance pressure. Temperature at which samples were injected was set at 230 °C, with an initial mold temperature of 55 °C. The maintenance time was 15 s. The injection process was based in one central injection point. 

**Figure 1:**
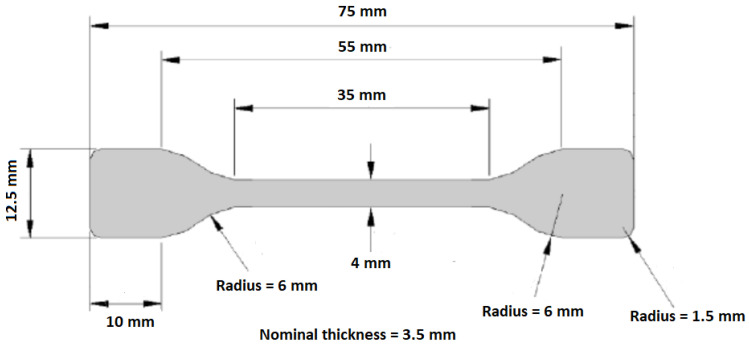
Sample mold’s nominal dimensions.

Due to material’s shrinkage, samples showed differences from nominal values in the values of the transversal width and thickness (Figure 1). Dimensions were reduced with glass fiber content. Width values measured showed values between 3.8 mm and 4.2 mm, while thickness values showed values from 3.1 mm to 3.9 mm in all samples injected, where samples of three materials were considered. After injection process, samples were trimmed in order to be adapted to the dynamic mechanical analysis (DMA). For this purpose, only the 30-mm central part of samples was considered. Depending on the test analysis carried, out one or two ends were clamped in DMA equipment.

**Figure 2:**
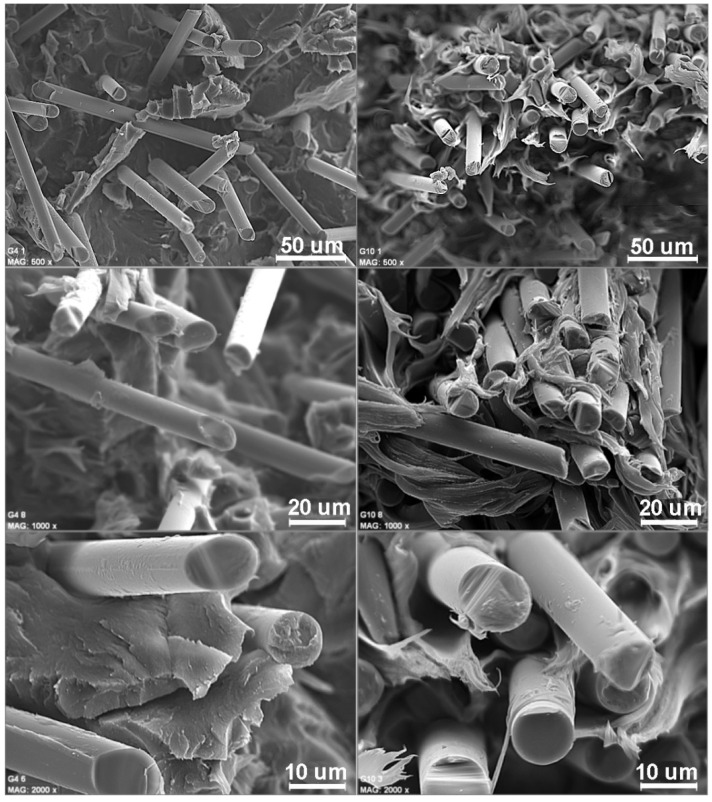
Scanning electron microscopy (SEM) of samples of PBT B 4300 G4 (left side) and PBT B 4300 G10 (right side) with magnitude amplification (500×, 1000×, and 2000×).

The material injected was observed by means of scanning electron microscopy (SEM, Hitachi, Chiyoda, Tokio, Japón). Fibers showed a random and distributed dispersion. In the following figures, photographs of samples of PBT G4 (left side) and PBT G10 (right side) are shown in three different amplification levels (Figure 2).

### 2.2. Dynamic Mechanical Analysis (DMA) and Sample Preparation

Tests were carried out in Q800 equipment (TA instrument, New Castle, DE, USA). 

#### 2.2.1. Dynamic Moduli and Glass Transition Temperature Evolution with the Temperature

The evolution of both the storage and loss moduli was studied in the multifrequency mode by maintaining frequency constant and provoking a temperature gradient. The glass transition temperature was determined with the maximum loss modulus. Single cantilever configuration was considered and subjected to a 0.1% strain increment and 1-Hz frequency. 

Tests were carried out at a range of temperatures from 25 °C to 225 °C with a 3 °C/minute temperature gradient, which allowed us to measure the evolution of both the storage and loss moduli for this whole temperature range.

#### 2.2.2. Multifrequency Assays

Multifrequency assays were performed between a range of 0.1 and 10 Hz at constant temperature. A work range between 25 °C and 145 °C was used with 3 °C intervals. Samples built in on one side were considered with a 10-μm amplitude and a 5-min temperature stabilization time. The variation in the glass transition temperature with frequency was evaluated by the maximums of the loss modulus curves, which were generated for the distinct frequencies by the TA Universal Analysis 2000 software (TA instrument). Master curves were generated with the TTS Data Analysis (TA instrument), and the applied temperature was that at which the maximum loss modulus appeared. Double cantilever configuration was considered for test development.

## 3. Analyzing and Discussing the Results

The results performed in the characterization tests of the Ultradur^®^ material in the studied configurations are shown below, considering the following notations:

- Ultradur^®^ B 4520: PBT GF0,
- Ultradur^®^ B 4300 G4: PBT GF20, and
- Ultradur^®^ B 4300 G10: PBT GF50.

### 3.1. Evolution of Dynamic Moduli with Temperature

**Figure 3:**
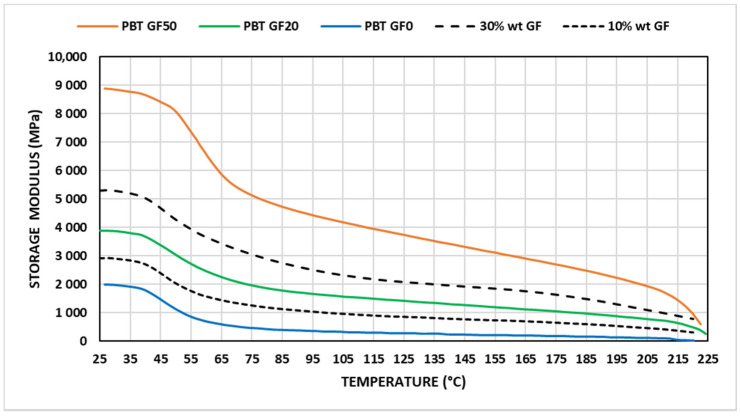
Variation in the storage modulus with temperature (f = 1 Hz).

The storage modulus showed a nonlinear behavior with temperature and drastically dropped at around the glass transition temperature (T_g_), as can be observed in Figure 3, which agrees with results shown by other authors [40,41,42]. At temperatures above T_g_, relatively large segments of 10 to 50 repeat units can move freely by changing their conformation. Below T_g_, these polymers have many properties associated with crystalline materials, while above T_g_, materials behave likely elastomers. In all ranges of temperatures analyzed, the behavior improved with glass fiber content. The reason is that glass fibers’ addition modified the structural behavior of the material that behaved like a composite material [33]. Near to the polymer melting temperature the reinforcement’s effectiveness was reduced. This behavior can be attributed to the fact that storage modulus depended on polymer’s properties, reinforcement’s properties, and interface’s properties between materials. Final value reached depended on materials’ percentage and temperature. Near melting temperature, interface properties were weakened, working the materials separately again.

Mortazavian and Fatemi [33] distinguished among three evolution intervals for the storage modulus with temperature in a simplified manner. In this work, for the rage of temperature analyzed, the storage modulus evolution was depicted by means of polynomial functions considering a modified mixture law and considering glass fiber content, according to the following equation.

(1) $$E^{\prime }=\eta \cdot \frac{\Phi_{f}\left(\%\right)}{100}\cdot E_{f}+\left(1-\frac{\Phi_{f}\;\left(\%\right)}{100}\right)\cdot {E^{\prime }}_{\mathrm{PBT}}\;(\mathrm{Units}\;\mathrm{MPa})$$

where E’(MPa) represents the storage modulus of composite material, E_f_ (MPa) represents the glass fiber modulus, E’_PBT_ (MPa) represents the storage modulus of the PBT matrix, Φ_f_ represents the fiber volume as a percentage, and η is a nondimensional parameter that denotes the reinforcement effectiveness factor. The effectiveness factor depends on fibers’ length, their degree of dispersion, and the effect of temperature on the adhesion force between fibers and matrix, which worsens with temperature. This work contemplated a temperature-dependent effectiveness factor based on a five-order polynomial, in which the parameters defining this polynomial presented linear dependence with the fiber volume expressed as a percentage.

(2) $$\eta \left(\Phi_{f},T\right)=\overset{i=5}{\underset{i=0}{\sum }}F_{i}\left(\Phi_{f}\right)\cdot T^{i}$$

(3) $$F_{i}\left(\Phi_{f}\right)=A_{i}\cdot \Phi_{f}\left(\%\right)+B_{i}\;(i\;=\;1\;\mathrm{to}\;5)$$

The values of the constants for a test run at a 1-Hz frequency appear in Table 1, where A_i_, B_i_, and F_i_ (i = 1 to 5) are expressed in the same units. The storage modulus evolution for glass fiber weight percentage of 10% and 30% in accordance with these parameters is reflected in Figure 3.

**Figure 4:**
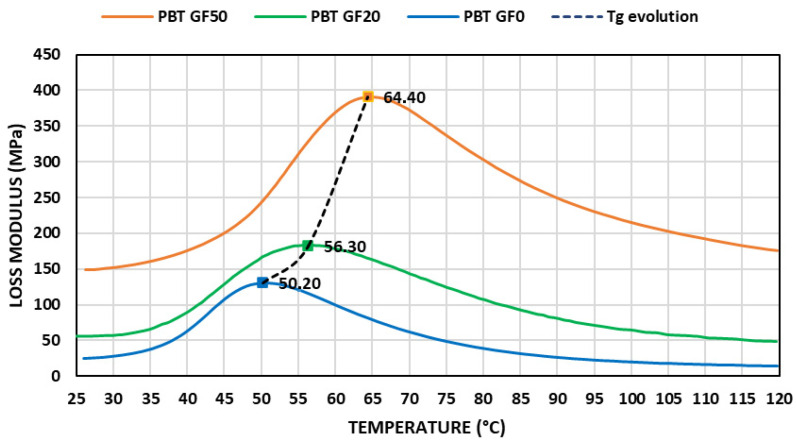
Variation in the loss modulus with temperature (f = 1 Hz).

Loss modulus showed a bell shape evolution with temperature, as can be observed in Figure 4. Maximum value was obtained at glass transition temperature. In all ranges of temperatures analyzed, the behavior improved with glass fiber content. This factor can be attributed to an increase in inner friction, allowing us to improve the energy dissipated in each strain cycle. 

Maximum peak and its amplitude increased with glass fiber content. This fact can be attributed to inhibited relaxation mechanisms on the interface between fibers and polymer matrix. Fibers induced constraints, imposing mobility restrictions in polymer chains at relaxation temperatures. Percentage of polymer chain immobilized increased with fiber content increasing the glass transition temperature obtained. With unreinforced material, the obtained values correlated well with other values found in the literature [43]. In this work a linear relationship between the glass transition temperature, considering maximum values of loss modulus, and the volumetric glass fiber percentage was considered (Table 2), according to the following equation:

(4) $$T_{g}\left({}^{\circ}C\right)=A+B\cdot \Phi_{f}\left(\%\right)$$

### 3.2. Glass Transition Temperature Evolution with Frequency

**Figure 5:**
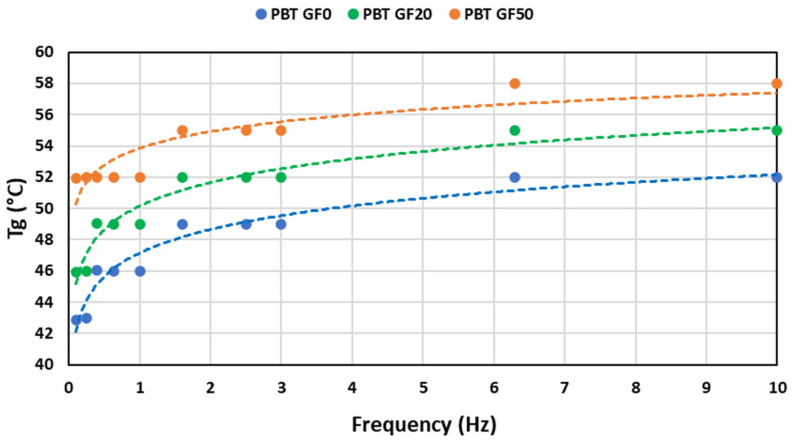
Variation in the glass transition temperature with frequency.

Glass transition temperature increased with frequency, as can be observed in Figure 5 where an asymptotic behavior in the glass transition temperature with frequency was observed, indicating a limit value of glass transition temperature (T_g_). At low frequencies, macroscopic deformation times came close to the order of magnitude of the microscopic deformation times, and dissipated energy increased by internal friction. When the strain rate was increased, polymer chains were less prone to these relaxation mechanisms, which resulted in those chains with shorter effective lengths supporting the load. The material showed less ductility and offered more resistance and a higher storage modulus. Variation in the storage modulus with frequency presented logarithmic evolution with the strain rate, according to several authors [40,41,42]. Glass transition temperature (T_g_) can be linearly fitted to the natural logarithm of frequency (f [Hz]) with very good regression coefficients, according to the following equation:

(5) $$T_{g}\left({}^{\circ}C\right)=C+D\cdot \ln\left(f\right)$$

where the defining parameters were obtained from test curves’ fitting and can be observed in Table 3. 

The difference in T_g_ found in the multifrequency test when frequency equaled 1 Hz, compared to the test with a constant frequency at 1 Hz, can be attributed to the differences in testing conditions. This emphasizes that T_g_ value was not a unique value. The value obtained depended on the technique, testing conditions, and methodology. The values showed a difference that was increased with glass fiber content, with a maximum difference of 19.5% for the samples reinforced with the 50% glass fiber weight. Glass transition temperature was linear with glass fiber content and the logarithm of the frequency, according to Equations (4) and (5). The values obtained could be combined considering the mean values between both equations for the 1-Hz frequency and by maintaining the T_g_ ratio for the other frequencies to calculate the mean values. Glass transition temperature could be obtained according to the following equation where parameters were obtained from Table 4.

(6) $$T_{g}\left({}^{\circ}C\right)=T_{g0}+K_{1}\cdot \Phi_{f}\left(\%\right)+K_{2}\cdot \ln\left(f\right)$$

The values obtained with the parameters in Table 4 presented an average difference of 5.45 ± 2.6% and 6.54 ± 3.8% in relation to the test values at the 1-Hz frequency and the multifrequency, respectively.

### 3.3. Master Curves

**Figure 6:**
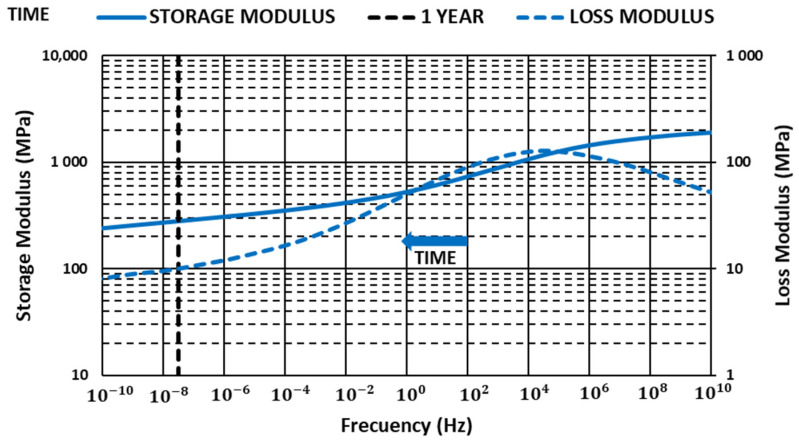
Variation in the storage and loss modulus with time PBT G0 (T = 50.2 °C).

**Figure 7:**
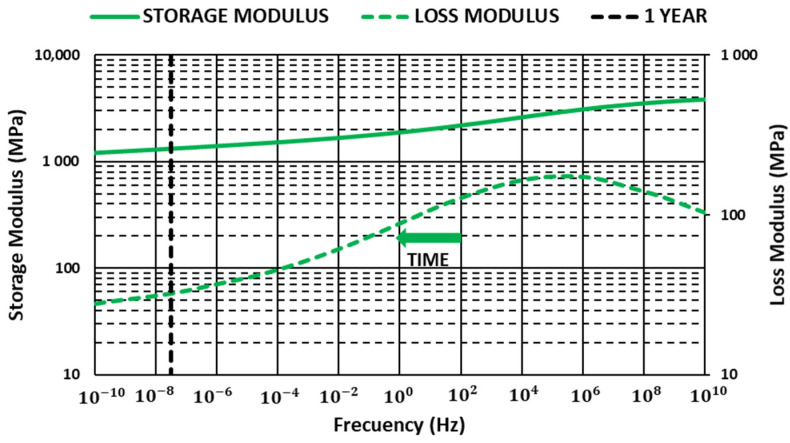
Variation in the storage and loss modulus with time PBT G20 (T = 56.3 °C).

**Figure 8:**
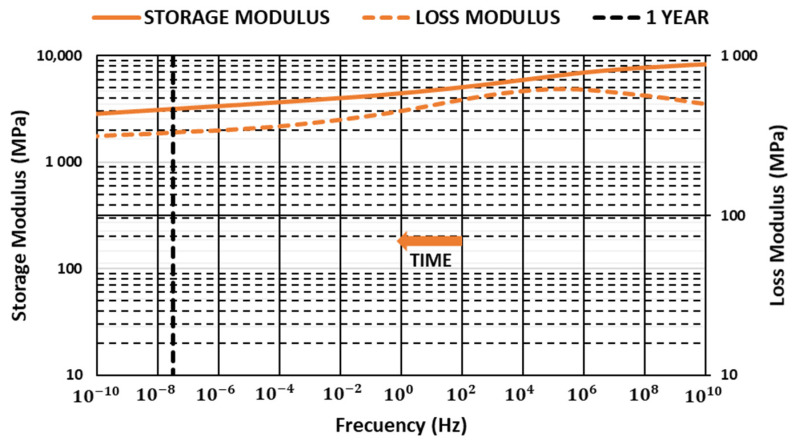
Variation in the storage and loss modulus with time PBT G50 (T = 64.4 °C).

The Data Analysis software of TA Instrument was used to produce master curves [44]. For this work, reference temperatures that were lower but close to the glass transition temperature were selected. Values were obtained in the tests run with a constant 1-Hz frequency for each material (Figure 6, Figure 7 and Figure 8). 

Storage modulus values showed a loss of stiffness properties over time that was mitigated with glass fiber content, allowing us to improve storage modulus in all ranges of frequencies analyzed. The results showed how glass fiber content can be useful to guarantee a minimum storage modulus value over time when components are submitted to high-temperature environments, allowing optimized and reduced polymer consumption.

Loss modulus increased with glass fiber content in all ranges of frequencies analyzed. Maximum value moved toward higher frequencies, decreasing the slope after the maximum, increasing in this way the dissipated energy.

**Figure 9:**
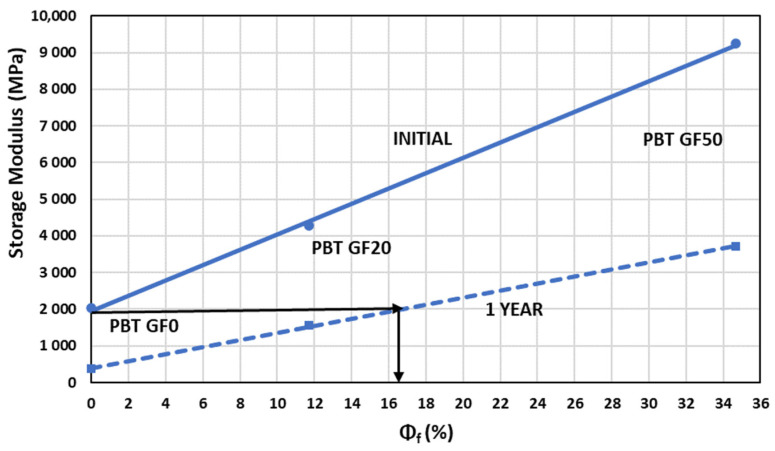
Variation in the storage modulus with the percentage of fiber volume depending on the exposure status.

The storage modulus value showed a linear behavior with glass fiber content when samples were held at a temperature that came close to the material’s glass transition temperature, bearing in mind its fiber percentage according to the following Figure 9:

The evolution with glass fiber content, expressed as volume percentage, for specimens initially exposed to their glass transition temperatures and after one year at temperatures close to said temperature presented a linear evolution given by the following equation, where parameters were obtained from Table 5:

(7) $$\;E^{\prime }=A\cdot \Phi_{f}\left(\%\right)+B\;\left(\mathrm{Units}\;\mathrm{MPa}\right)$$

The above equation allowed us to choose the fiber content percentage in order to maintain the value of PBT storage modulus over time at one temperature higher to the glass transition temperature of this material. For example, in the figure above, with PBT reinforced with glass fiber in a volume percentage near to 16% (equivalent to a weight percentage near to 30%), a minimum storage modulus greater or equal to PBT without reinforcement can be guaranteed for one year, when the sample is held to a temperature of 57.2 °C (obtained with Equation (4)).

## 4. Conclusions

PBT is a technical plastic that performs extremely well as an electric insulator with very few dielectric losses. Its use has considerably increased thanks to a growing number of electronic and electric systems. It is normally manufactured by incorporating glass fiber to improve mechanical properties and can also be blended with elastomeric materials to improve its performance upon impacts and fracture tenacity. Furthermore, PBT offers extremely good versatility, which has enabled additives to be incorporated, such as flame retardants and, particularly, more enviro-friendly nonhalogen additives, to improve their properties against fire. All these advantages make PBT an ideal candidate to develop new electrical components associated with charges or battery in all sectors and particularly in the growing transport sector.

The results of this work showed the evolution over time of dynamic moduli of PBT reinforced with glass fiber when samples were submitted to high-temperature environment.

The current study demonstrated that glass fibers’ addition allowed us to increase the value of dynamic moduli in all ranges of temperatures analyzed, allowing us to reduce the loss of stiffness over time and increasing the energy dissipated in each strain cycle.

The dynamic moduli evolution with time was obtained by means of a temperature–time transformation using the Data Analysis software of TA instrument and considering glass transition temperature obtained from maximum of loss modulus with temperature. Glass transition temperature increased linearly with glass fiber content and logarithm of the frequency, indicating a limit value of T_g_ with frequency.

Dynamic moduli showed a nonlinear evolution with time when samples were hold to temperatures lower but close to their glass transition temperature. The values obtained improved with glass fiber content in all ranges of frequencies analyzed. In this work, a linear fit of storage modulus depending on fiber content, when samples are submitted to their respective glass transition temperature, was proposed, allowing us to choose the fiber content percentage in order to maintain a value greater than or equal to PBT storage modulus over time at one temperature higher to the glass transition temperature of this material.

## Figures and Tables

**Table 1:** Constants defining the effectiveness factor (f = 1 Hz).

Constants for Polynomial Coefficients	A_5_/B_5_ (°C^−5^)	A_4_/B_4_ (°C^−4^)	A_3_/B_3_ (°C^−3^)	A_2_/B_2_ (°C^−2^)	A_1_/B_1_ (°C^−1^)	A_0_/B_0_ (-)
A value	2.37 × 10^−13^	−1.55 × 10^−10^	3.62 × 10^−8^	−3.59 × 10^−6^	1.17 × 10^−4^	1.19 × 10^−3^
B value	6.74 × 10^−12^	−4.64 × 10^−9^	1.17 × 10^−6^	−1.31 × 10^−4^	5.31 × 10^−3^	1.56 × 10^−1^

**Table 2:** Defining parameters of T_g_ evolution (°C) with the glass fiber volume.

A (°C)	B (°C)	R^2^
50.76	0.4	0.989

**Table 3:** Defining parameters of T_g_ evolution (°C) with frequency.

Material/Parameters	C (°C)	D (°C/ln (Hz))	**R^2^**
PBT GF0	47.15	2.18	0.94
PBT GF20	50.15	2.17	0.94
PBT GF50	53.85	1.54	0.81

**Table 4:** Defining parameters of T_g_ evolution (°C) with the glass fiber volume percentage and frequency.

T_g0_ (°C)	K_1_ (°C)	K_2_ (°C/ln (Hz))
48.955	0.31	2.04

**Table 5:** Defining parameters of the storage modulus evolution with the percentage of glass fiber volume depending on exposure time.

Exposure Time/Parameters	A (MPa)	B (MPa)	**R^2^**
INITIAL	209	1955	0.99
1 YEAR	95.8	397.2	0.99

## Data Availability

Data available on request due to restrictions eg privacy or ethical.
